# Comprehensive Preterm Breast Milk Metabotype Associated with Optimal Infant Early Growth Pattern

**DOI:** 10.3390/nu11030528

**Published:** 2019-02-28

**Authors:** Marie-Cécile Alexandre-Gouabau, Thomas Moyon, Agnès David-Sochard, François Fenaille, Sophie Cholet, Anne-Lise Royer, Yann Guitton, Hélène Billard, Dominique Darmaun, Jean-Christophe Rozé, Clair-Yves Boquien

**Affiliations:** 1 INRA, UMR1280, Physiopathologie des Adaptations Nutritionnelles, Institut des maladies de l’appareil digestif (IMAD), Centre de Recherche en Nutrition Humaine Ouest (CRNH), Nantes F-44093, France; Thomas.Moyon@univ-nantes.fr (T.M.); agnes.david@univ-nantes.fr (A.D.-S.); helene.billard@univ-nantes.fr (H.B.); dominique.darmaun@chu-nantes.fr (D.D.); jeanchristophe.roze@chu-nantes.fr (J.-C.R.); clair-yves.boquien@univ-nantes.fr (C.-Y.B.); 2 Service de Pharmacologie et d’Immunoanalyse, Laboratoire d’Etude du Métabolisme des Médicaments, CEA, INRA, Université Paris Saclay, MetaboHUB, F-91191 Gif-sur-Yvette, France; francois.fenaille@cea.fr (F.F.); sophie.cholet@cea.fr (S.C.); 3 LUNAM Université, ON;IRIS, Laboratoire d’Etude des Résidus et Contaminants dans les Aliments (LABERCA), USC INRA 1329, Nantes F-44307, France; anne-lise.royer@oniris-nantes.fr (A.-L.R.); yann.guitton@oniris-nantes.fr (Y.G.); 4 CHU, Centre Hospitalo-Universitaire Hôtel-Dieu, Nantes F-44093, France; 5 EMBA, European Milk Bank Association, Milano I-20126, Italy

**Keywords:** breast milk metabolome, glycome, lipidome, free amino acid, preterm infant, growth trajectory

## Abstract

Early nutrition impacts preterm infant early growth rate and brain development but can have long lasting effects as well. Although human milk is the gold standard for feeding new born full-term and preterm infants, little is known about the effects of its bioactive compounds on breastfed preterm infants’ growth outcomes. This study aims to determine whether breast milk metabolome, glycome, lipidome, and free-amino acids profiles analyzed by liquid chromatography-mass spectrometry had any impact on the early growth pattern of preterm infants. The study population consisted of the top tercile-*Z* score change in their weight between birth and hospital discharge (“faster grow”, *n* = 11) and lowest tercile (“slower grow”, *n* = 15) from a cohort of 138 premature infants (27–34 weeks gestation). This holistic approach combined with stringent clustering or classification statistical methods aims to discriminate groups of milks phenotype and identify specific metabolites associated with early growth of preterm infants. Their predictive reliability as biomarkers of infant growth was assessed using multiple linear regression and taking into account confounding clinical factors. Breast-milk associated with fast growth contained more branched-chain and insulino-trophic amino acid, lacto-N-fucopentaose, choline, and hydroxybutyrate, pointing to the critical role of energy utilization, protein synthesis, oxidative status, and gut epithelial cell maturity in prematurity.

## 1. Introduction

A growing body of evidence supports the impacts on lifelong health of exposure to multiple factors in early life [1]. Therefore, studying the influence of intrauterine environments and perinatal exposure are keys to understanding early growth and development and health throughout life. Indeed, putative benefits of breastfeeding in new born full-term infants are, at least in part, due to its complex composition in various macronutrients, micronutrients, and other bioactive compounds [2,3,4]. Maternal breast milk is the recommended nutrition for feeding pre-mature infants [5], due to its reported health benefits such as (i) a significant decrease in the risk of developing prematurity-related morbidities [6,7], including necrotizing enterocolitis [8] and infection [8,9]; (ii) a significant decrease in the feeding intolerance [8,10]; and (iii) an improvement in neurodevelopmental outcomes [8,11]. However, feeding unfortified human milk may lead to insufficient or inadequate postnatal nutritional intake for many preterm infants in the first few weeks of extra-uterine life, particularly the very preterm infants born with a low birth weight and before 28 weeks gestation. Additionally, it is often associated with extra-uterine growth restriction [11,12], which could have severe adverse consequences in term of developmental delay [13,14,15]. Fortification of human milk is therefore recommended by the European Society for Paediatric Gastroenterology Hepatology and Nutrition (EPSGHAN) [16]. Yet, even among preterm infants receiving protein-fortified human milk, a large range of variation is observed in the early postnatal growth patterns [17].

The host of low-molecular-weight metabolites present in breast milk fully justifies the application of metabolomics/lipidomics, a promising holistic approach in neonatology used, by our [18,19,20] and other laboratories [21,22,23]. Metabolomics have been shown to generate new insights when investigating human milk [20,24,25,26] during the first month of lactation [27] or pre-term and full-term human milk metabolomes over a full lactation period [28]. We also reported, for the first time, the association of early growth trajectory with a specific lipidomic signature in the human milk of mothers delivering preterm infants over the first month of lactation [20]. Human milk oligosaccharides (HMO) are other unique components known to affect the gut microbiota and may contribute to the reduced incidence of necrotizing enterocolitis [28,29,30], improved brain development [31], and growth patterns observed in breastfed infants [32,33]. Additionally, amino acid [34] and fatty acid [35] metabolism by mammary gland were suggested to affect milk production and infant growth, leading to a metabolic imprinting, which may persist into adulthood. To the best of our knowledge, this is the first study to explore in depth the relationships between the metabolome, lipidome, and glycome of human milk, and the early preterm infant growth during hospital stays in neonatal intensive care units. To fill this gap, we tested the potential of the liquid chromatography-mass spectrometry-based phenotypic approach to investigate the composition of human breast milk from mothers delivering a preterm newborn during the early course of lactation. More in detail, the current study aims at shedding light on the relationships between breast milk composition, characterized using targeted free amino acid pattern and non-targeted metabolomic, lipidomics and glycomic signatures, and the early growth of preterm infants nourished by their own mother’s milk. As in our earlier reported pilot study [20], the present work was conducted within a larger prospective-monocentric-observational early birth LACTACOL cohort in which we selected two groups of preterm infants presenting very different growth trajectories during hospital stays. We previously reported in details [20] the breast milk lipidome in link with both infant growth groups, during the first month of lactation. The aims of the present work therefore are three-fold: (i) to assess metabolome, glycome and free amino acids pattern in the breast milk provided to preterms infants from week two to week four of lactation; (ii) to evaluate, initially and in week three-expressed breast milk samples, the interactions between human breast milk metabolome, lipidome and glycome and their association with the weight gain of infants between birth and time of discharge; and (iii) to identify a set of breast milk biomarkers with predictive ability on the postnatal weight growth trajectory of the preterm infants, taking into account confounding clinical factors. We hypothesized that our holistic approach, incorporating data from multiple breast milk compartments (i.e., metabolome, glycome, lipidome, and free amino acids), would considerably enhance our understanding of the molecular mechanisms linking breast milk composition to optimal early-growth of preterm infants.

## 2. Materials and Methods

### 2.1. Study Design and Population

**Figure 1:**
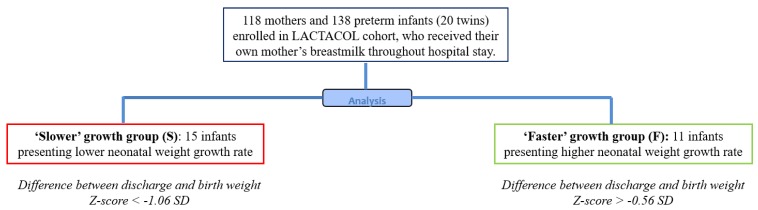
Study flowchart of infants enrolled in the ancillary study of the mono-centric prospective population-based LACTACOL (for global study flowchart of LACTACOL, see [20]). Among the 138 infants included in the LACTACOL cohort, no infant presented necrotizing enterocolitis (NEC), 4 infants had retinopathy of prematurity (ROP) of light severity, 3 presented intraventricular hemorrhage (IVH) of grade 2, 8 displayed bronchopulmonary dysplasia (BPD) at 28 days and 6 at 36 weeks’ postmenstrual age. The 26 selected infants did not have NEC, ROP, or BPD.

The present pilot study was conducted within a larger prospective study of the previously published LACTACOL birth cohort of preterm infant mother dyads [20], whose primary objective was to explore the impact of breast milk protein content received by preterm infants during hospital stays, on neurodevelopmental outcomes at 2 years of age. A total of 118 mothers and 138 infants born between 27–34 weeks of gestational age with no severe congenital pathology and no major diseases, except prematurity and who received, for a minimum of 28 days, their own mother’s breast milk only, were finally enrolled in the LACTACOL cohort (Figure 1). The current data were obtained on both sub-groups of infants selected among infants enrolled in the LACTACOL cohort and in the ancillary study, whose aim was to assess the relationship between breast milk composition (metabolome, lipidome, glycome, and amino acids) and preterm infant’s growth pattern during the first month of life. These 26 selected infants presented no severe neonatal morbidity or necrotizing enterocolitis or retinopathy of prematurity.

Clinical characteristics were collected both on mothers and infants, including: maternal age, educational level, pre-gravid body mass index (BMI), adverse events during pregnancy and delivery, infants’ gestational ages, birth weight, and head circumference, growth trajectory through hospital discharge, and events during hospital stays in neonatology. According to the EPSGHAN recommendations [16], preterm infants received parenteral nutrition and minimal enteral feeding with expressed breast milk predominantly provided by their own mother and fortified using Eoprotine^®^ (Milupa, 1564 Domdidier, Suisse) and FortiPré^®^ (Guigoz, 77186 NOISIEL, France) for protein and carbohydrate intakes, and Liquigen^®^ (Nutricia, 93406 Saint-Ouen, France) for lipid intakes, as previously detailed [20].

### 2.2. Ranking Infants according to Early Growth Trajectory

Infants enrolled in the LACTACOL cohort were ranked according to their change in weight *Z*-score (expressed in units of Standard Deviation (SD) and calculated as previously described [20] between birth and hospital discharge). For the first time, we chose to limit our longitudinal analysis of human breast milk composition to a small number of preterm mother infant dyads with no formal sample size calculation due to the exploratory nature of this pilot study. Then, the present study population consisted of the top tercile-*Z* score change in their weight between birth and hospital discharge (“faster grow”, *n* = 11 infants) and lowest tercile (“slower grow”, *n* = 15 infants), from our population of 138 enrolled preterm babies (born before 32 weeks gestation) (Figure 1).

### 2.3. Ethics

This research study was approved by the National Data Protection Authority (Commission Nationale de l’Informatique et des Libertés, N° 8911009) and the appropriate ethics Committee for the Protection of People Participating in Biomedical Research (CPP-Ouest I, reference CPP RCB-2011-AOO292-39). The current data were obtained in the ancillary study number three of the LACTACOL cohort registered at www: clinicaltrials.gov under #NCT01493063. The milk biobank was approved by the Committee for the Protection of Persons in medical research (CPP CB-2010-03). Parents received oral and written information in the maternity ward or neonatal unit and lactation support and training on proper sample collection from the study lactation consultant. A written consent was obtained from all parents at enrolment.

### 2.4. Human Milk Collection and Targeted Free Amino Acid (FAA) Analysis

Weekly representative 24-h breast milk expression was performed manually by mothers at home during all the lactation periods corresponding to their infant hospital stay, then processed, aliquoted, and frozen at −80 °C until analysis, as previously described [20]. FAA concentrations were determined in expressed breast milk samples collected from week two to week four of lactation using Ultra Performance Liquid Chromatography-High-Resolution-Mass Spectrometry (UPLC-HR-MS), as previously described [36]. Briefly, following a delipidation step by centrifugation and a deproteinization step by addition of sulfosalicylic acid and centrifugation, free amino acids (FAA) from supernatant were derivatized using AccQ^®^TagTM Ultra reagent (Waters Corporation, Milford, MA, USA)), separated on an Acquity H-Class^®^ UPLC system (Waters Corporation, Milford, MA, USA), combined with a Xevo TQD^®^ mass spectrometer (Waters Corporation, Milford, MA, USA), then identified and quantified, using the Waters TargetLinks^TM^ software (Waters Corporation, Milford, MA, USA).

### 2.5. Breast Milk Liquid Chromatography-High-Resolution-Mass Spectrometry (LC-HRMS)–Based Glycomic Profiling

The extraction and reduction of oligosaccharides in human milk collected from week two to week four of lactation were performed as previously described [37]. Briefly, 10 µL of human milk were diluted by adding 450 µL of water and then delipidated by centrifugation. The lower phase was reduced with an NaBH4 solution and loaded onto a porous graphitized PGC cartridge (Hyperseb Hypercarb^®^, Thermo Scientific, San Jose, CA, USA). Milk reduced oligosaccharides were separated on a Hypercarb^®^ column (2.1 mm i.d. × 100 mm, 3 µm particle size, Thermo Scientific, San Jose, CA, USA) on an Ultimate 3000 HPLC system (Thermo Scientific, San Jose, CA, USA). HMO chromatographic separation was performed at 30 °C with a flow rate of 300 µL/min using the gradient conditions with mobile phases A (water containing 0.1% formic acid) and B (acetonitrile containing 0.1% formic acid), as described by Oursel et al. [37]. Column effluent was directly introduced into the electrospray source of a hybrid quadruple time-of-flight (Q-TOF Impact HD) instrument (Bruker Daltonics, Bremen, Germany) operating in the positive ion mode. The source parameters were the following: 3700 V for the capillary voltage, 8.0 L/min for the dry gas, and 200 °C for the dry heater.

### 2.6. Breast Milk Liquid Chromatography-High-Resolution-Mass Spectrometry (LC-HRMS)–Based Lipidomic and Metabolomic Profiling

The organic and aqueous layers, following Bligh-Dyer extraction [38] of the same milk samples, were collected from week two to week four of lactation, dried separately, and subsequently reconstituted in acetonitrile-isopropanol-water (ACN: IPA: H_2_O 65:30:5, *v*/*v*/*v*) and in water-acetonitrile (H_2_O: ACN 95:5, *v*/*v*) for lipid and polar species, respectively. Then, lipidomic and metabolomic profilings were performed using separation on a 1200 infinity series^®^ HPLC-system (Agilent Technologies, Santa Clara, CA, USA) coupled to an Exactive Orbitrap^®^ MS (Thermo Fisher Scientific, Bremen, Germany) equipped with a heated electrospray (H-ESI II) source (operating in polarity switch mode), as previously described [20]. Concerning lipidomic profiling, a reverse phase CSH^®^ C_18_ (100 × 2.1 mm^2^ i.d., 1.7 µm particle size) column (Waters Corporation, Milford, MA, USA) was used for lipid species separation using ACN:H_2_O (60:40) and IPA:ACN:H_2_O (88:10:2) as solvent A and B, respectively, with both containing 10 mM ammonium acetate and 0.1% acetic acid [39]. Concerning metabolomics fingerprinting, polar species separation was performed on the same LC-HRMS system on a reverse phase with a Hypersil GOLD C18 column (1.9 μm particle size, 100 × 2.1 mm) using a mobile phase of water (95%) and acetonitrile (5%), each containing 0.1% acetic acid according to Courant et al. [40]. The precision associated with sample preparation and LC-HRMS measurement was determined on the basis of a quality control (QC) consisting of a pool of 10 mothers’ milk provided by the milk bank of Nantes Hospital Center.

### 2.7. Lipidomic, Metabolomic and Glycomic Data Treatment and Metabolites Annotation

Lipidomics and metabolomics raw data files were preprocessed and converted to the *.mzXML open file format using Xcalibur 2.2^®^ (Thermo Fisher Scientific, San Jose, CA, USA) and MSConvert^®^ (http://proteowizard.sourceforge.net/), respectively [41]. Then, lipidomics and metabolomics data were extracted using (i) pre-processing with the open-source XCMS^®^ [42] within Workflow4Metabolomics^®^ (W4M) (http://workflow4metabolomics.org) [43] for nonlinear retention time alignment and automatic integration for each detected features combined with CAMERA^®^ [44] for annotation of isotopes and adducts, and (ii) normalization of intra- and inter-batch effects using Quality Control (QC) samples [45]. A manual curation, for the quality of integration and a filtration of the resulting XCMS (m/z; Retention Time (RT)) features by a 30% relative SD cutoff within the repeated pooled QC injections [46] were performed. Thereafter, accurate mass measurement of each putative metabolite was submitted to LIPID Metabolites and Pathways Strategy (LipidMaps^®^, www.lipidmaps.org), Human Metabolite Data base (HMDB^®^, www.hmdb.ca), Biofluid Metabolites Database (MetLin^®^, metlin.scripps.edu), and Milk Metabolome Database (MCDB^®^, www.mcdb.ca) annotation. Moreover, the lipids and metabolites of interest were identified with the use of the (pseudo) tandem mass spectrometry spectrum generated by all ion fragmentation [39] combined with the use of in-house reference databanks [47]. Metabolite’s identification level was level one, for metabolites definitively annotated with our home data base (i.e., based upon characteristic physicochemical properties of a chemical reference standard (m/z, RT) and their M/MS spectra compared to those of breastmilk QC) or level two, for metabolites putatively annotated (i.e., without chemical reference standards, based upon physicochemical properties and MS/MS spectral similarity with public/commercial spectral libraries, e.g., LipidMaps^®^, MetLin^®^, and MCDB^®^). Monosaccharide compositions of HMOs were deduced from accurately measured masses (<5 ppm on average) and previously determined retention times were obtained through the use of some commercial HMO molecules [37]. Complementary MS/MS experiments were then performed to confirm putative structures. When it was not possible to clearly determine HMO structures, HMOs were named according to their monosaccharide compositions and denoted as hexose (Hex), fucose (Fuc), N-acetylhexosamine (HexNac), and N-acetylneuraminic acid (NeuAc) numbers. In addition, isomeric forms were distinguished by a lower-case letter added after the monosaccharide composition (e.g., 4230a and 4230b). Overall, 89 (45 monosaccharide compositions) distinct HMOs were detected. Relative HMO abundances were calculated by dividing absolute HMO peak area by each sample’s total HMO peak areas.

### 2.8. Statistical Analyses

In Table 1, Table 2, Table 3 and Table 4, values were reported as medians and 25% and 75% percentiles. Statistical analyses were carried out using GraphPad Prism^®^ software version 6.00 (La Joya, CA, USA), SIMCA P^®^ version 14 (Umetrics AB, Sweden) and R version 3.4. (R Development Core Team, 2013; http://www.R-project.org). For all data analyses, the significance level (α) was set to 5%. Multivariate statistical models were applied separately on each glycomic, lipidomic, metabolomics, fatty acids and free amino acids data matrix considering the *a priori* structure into “faster” vs. “slower” infants’ growth groups. We chose to take into account the higher (compared to glycomic data) variability in magnitude for lipidomic and metabolomic features; this is the reason why a Log Pareto scaling [48] was performed. Lipidomic or metabolomic data were submitted to the statistical workflow previously used with success on lipidomic profiling [20] in order to: (i) select the lipid/metabolic species providing a clear separation between the two infant postnatal growth subgroups from week two to week four of lactation, using the Analysis of Variance-PLS (AoV-PLS) combined with a Fisher’s Linear Discriminant Analysis (LDA) procedure [49]; (ii) check the selected biomarkers predictive ability for infant weight growth, using Mann-Whitney *U*-test combined with multiple testing filtering (FDR); and (iii) confront them to the various confound clinical variables (mother’s body mass index, birth weight, gestational age, complementary parenteral and enteral nutrition with the protein, lipid and energy intakes, duration of parenteral feeding and ventilation, and length of hospital-stay) and, in turn, test their reliability as biomarkers of infant’s growth, by using multiple linear regression (MLR) combined to FDR on the remaining variables candidates as biomarkers, i.e., 80, 60, or 35 models for metabolomic, lipidomic or glycomic data, respectively. Moreover, we hypothesized that high-level data fusion, resulting in a meaningful synthesis, was expected to provide a holistic picture of the preterm breast milk composition. In order to integrate multiple–omics analytical sources and chemometrics for a comprehensive metabolic profiling of human preterm milk associated with an optimal infant weight growth, we used clustering or classification methods aiming at discriminating groups of milks using “omics” data. In order to simplify the model, we discarded the time lactation point factor of the present study and focused on “omics” data provided at week three of lactation, which had previously been shown to display the higher discriminating effect on preterm breast milk lipidome [20]. Additionally, as including an excessive amount of irrelevant variables would deteriorate the models, and in order to ovoid overfitting, all variables provided by AoV-PLS-DA scores with variables of importance in the projection (VIP)-index below 1.5 were removed in both metabolomic and lipidomic data and only the annotated representative metabolites and lipid species were kept. The input resulting metabolomic and lipidomic Log Pareto-scaled blocks were concatenated with mean and deviation standard-scaled blocks (i.e., glycomic profiling, fatty acid, and free amino acid patterns). Then, we tested on the super-matrix thus obtained an unsupervised unfold principal components analysis (UPCA-clustering method) [50] and supervised multi-block partial least squares analysis (MB-PLS- classification method) [51] strategies that searched for directions of similar sample distributions in the multidimensional spaces defined by each block of “omics” data, i.e., common components. Variables of interest for the discrimination of milk metabotype were selected according to their coordinates on the common components axes in the MB-PLS model.

## 3. Results

### 3.1. Subject Characteristics

The median difference between discharge and birth weight *Z*-score was −0.479 SD and −1.538 SD, for infants with “faster growth” and “slower growth”, respectively. Two sets of twins belonged to the “slower” growth group, and two others sets of twins followed opposite trajectories regarding their weight *Z*-score difference between birth and hospital discharge, i.e., one twin belonged to the “faster” growth group, whereas the other twin belonged to the “slower” growth group. Table 1 displays the median maternal and infants’ characteristics. Despite similar gestational age and hospital stay-lengths, the group of infants with ‘faster’ growth presented a 25% lower birth weight and a 69% greater gain in weight *Z*-score compared to the group of infants with “slower” growth. This negative correlation between birth weight and weight *Z*-score at time of discharge was previously reported in the large LIFT cohort of 2277 preterm infants by our team [52] and in another cohort [53].

Initially, time course breast milk compositional changes were detected, during the first month of lactation in our two sub-groups of 11 mothers delivering preterm newborns, who presented very different growth trajectories during their hospital stays using (i) targeted free amino-acids quantification combined with (ii) metabolomic (and lipidomic) and (iii) glycomic signatures. Then, multi- and univariate statistical models were applied to identify significant changes in metabolites that are associated with early postnatal infant growth. For second time, we focused (iv) our “omics” data fusion models on one representative time of lactation (week 3) to identify similar expression changes in various molecules and, in turn, highlight a few biological pathways of interest associated with optimal preterm infant growth.

### 3.2. Targeted Free Amino Acid Quantification

In the present pilot study, breast milk provided to the “faster” growth group presented a slightly higher essential amino acid content combined with a significantly higher content of branched-chain, insulinotrophic and gluconeogenic amino acids, as well as a decrease in sulfur amino acid content (with only taurine and methionine quantified). More specifically, breast milk arginine and tyrosine concentrations were significantly higher in the “faster” growth group than that in the “slower” growth group, whereas glycine and taurine levels were lower in the “faster” growth group with a trend toward lower glutamate and glutamine concentrations. Considering the predictive ability of free amino acid for infant weight growth during hospital stay, branched-chain and insulinotrophic amino acids were significant using multiple linear regression combined with multiple correction and taking into account maternal and infant clinical variables, whereas sulfur amino acids (taurine and methionine) presented only a trend (Table 2).

### 3.3. Lipidomics and Metabolomcs Profiling

**Figure 2:**
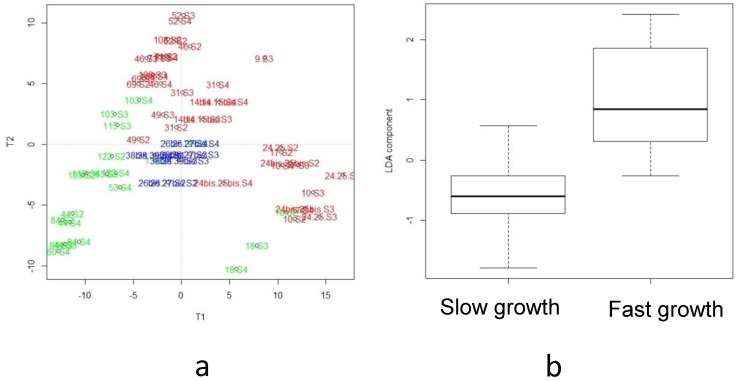
Analysis of Variance (AoV)-PLS and linear discriminant analysis (LDA) models based on the LC-ESI^+^-HRMS metabolomics profiles of human preterm milk on the factor weight *Z*-score (discharge-birth): AoV-PLS score plot with 56% of variance (R2Y = 34%) on components 1–2 (**a**) and LDA (built on components of AoV-PLS) with a *p*-value = 0) (**b**). Breast milk provided to preterm infants who experienced “faster” (green) or “slower” (red) growth and to twin infants with discordant growth rate, one twin with high growth rate and the other one with low growth rate, (blue).

Lipidomic analysis of the human breast milk provided (from week two to week four of lactation) to the 26 infants selected in the present pilot study was previously reported [20]. The most discriminant features associated with infant growth during hospital stays corresponded to a cluster of 1256 VIP-based lipid species. Among the 50 AoV-PLS/LDA- and FDR-selected annotated lipid biomarkers, nine lipid species appeared of paramount interest due to their significant (10% threshold) MLR q-value for delta weight *Z*-score (data from Table 4, [20]). Similarly, metabolomic LC-HRMS (ESI^+^/ESI^−^) data obtained on preterm breast milk from week two to week four of lactation, were processed using AoV-PLS procedure [49] to assess the association between the metabolites and the *a priori* grouping structure (“faster” vs. “slower” infant growth). The score plots clearly highlighted the separation between breast milk metabotypes associated with ‘faster’ or ‘slower’ infant growth in both positive (Figure 2a) and negative (supplementary Figure S1) ionization modes with the breast milk metabolomic profiles, corresponding to the four sets of twins plotted between both clusters (depicted with blue symbols in Figure 2a and Figure S1a). Then, the selected appropriate components of AoV-PLS (for both ionization modes) were subjected to a Fisher’s linear discriminant analysis (LDA) to test the significance of growth factor (Figure 2b and Figure S1b). Their cross-validation error rates of the LDA canonical variables for positive and negative mode were both equal to 7.14%. The most discriminant features associated with infant growth during hospital stay corresponded to a cluster of 125 (resp. 119) VIP-based metabolites species (VIP-index above 1.5) in the positive (resp. negative) ionization mode leading to 68 features that could be annotated (Table 3).

We identified (Table 3) the association between our two groups of preterm infant’s growth and breast milk metabolites (VIP in AoV-PLS models) that manually map to pathways such as the arginine-creatinine pathway, aromatic amino acid metabolism (including intermediate products of tryptophan, tyrosine, and/or phenylalanine catabolism), nicotinamide adenine dinucleotide precursors (such as nicotinamide and tryptophan), sulfur metabolism, oligosaccharides (e.g., 2’-Fucosyllactose and Lacto-N-FucoPentaose), mitochondrial fatty acid beta-oxidation, (including metabolites such as acylglycine and several analytes of the tricarboxylic acid cycle), pyruvic, citraconic, and aconitic acids, and choline metabolism. Many metabolites were significantly different between both groups of infants’ growth when their multiple comparisons adjusted *P*-values (i.e., *q*-value after false discovery rate (FDR)) was <0.1, such as higher levels in orotic acid, nicotinamide, hydroxybutyric acid, pyruvic and citraconic acids, and choline in the “faster” group besides lower abundance in cresol and benzoic acid, for example. Among these metabolites, only a few metabolites predicted early infant growth (significant MLR *p*-value but unsuccessful for multiple correction testing, FDR-corrected MLR *q*-value), including: hydroxy-3-methylbutyric acid, undecenal, dodecanedioic acid and choline.

### 3.4. Glycomics Profiling

**Figure 3:**
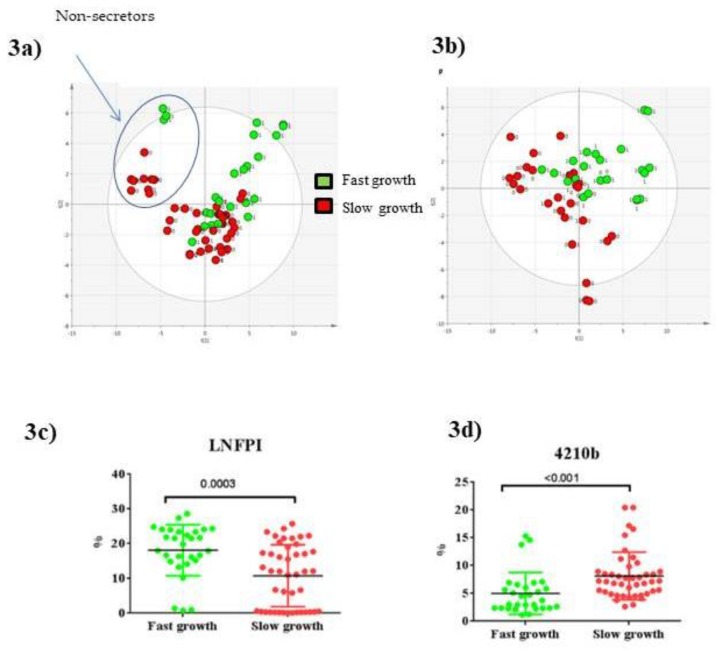
PLS-DA score plot based on the LC- HRMS glycomics profiles of human preterm milk of all mothers (**a**) (with non-secretor mothers circled on the basis of on the concentration of 2’-FL in their milks) or only of secretor mothers (**b**), on the factor weight *Z*-score (discharge-birth) with 45–39% of variance (R2Y = 37–45%), respectively, on components 1–2. Scatter plot (median) from W2 to W4 of lactation period (with secretor and non-secretor mothers) for two representative HMOs selected among VIP of interest: LNFPI (**c**) and 4210b (**d**), respectively. *p* values for comparison between “faster” and “slower” growth groups were derived using Mann-Whitney *U* test.

Milk samples were analyzed for changes in their HMO profiles from W2 to W4 of lactation in association with postnatal weight growth trajectory during hospital stay. On the PLS-DA score plot of PLS components (PCs 1 and 2) (Figure 3a), as expected, there was a clear difference in milk HMO profiles depending on secretor status in preterm milk samples, as the milk samples were separated into secretor (21 mothers) or non-secretor (five mothers) groups. The overal percentage of non-secretor mothers in our sub-cohort of LACTACOL was consistent with the proportions in most human populations, i.e., approximately 20% [28], but was significantly higher for mothers of infants ranked in the “slower” growth group (36%, i.e., 4 non-secretor mothers) versus those ranked in the “faster’’ growth group (10%, i.e., only one non-secretor mother). In this study, secretor or non-secretor status (i.e., mothers expressing or not the 1,2-fucosyl-transferase 2) was essentially defined by either high or low 2’Fucosyllactose (2’FL) levels, respectively, as measured by LC-MS [28,54]. Milk from mothers that was classified as non-secretors showed very low or even no detectable levels of 2’-FL, whereas milk from mothers classified as secretors contained high amounts of 2’-FL. Interestingly, exclusion of the five HMO profiles from non-secretor mothers in the statistical PLS-DA model improved the separation between the milks of the “faster” and “slower” infant growth groups (Figure 3b). As shown in Table 4, relative abundances of several measured HMOs differed significantly between breast milks provided to preterm infants with “faster” or “slower” growth during the W2 to W4 lactation period with higher overall levels of HMOs in milk given to fast-growing infants. In detail, and regardless of the maternal secretor status, breast milk provided to infants with optimal growth contained more total fucosylated HMOs (essentially due to the mono-fucosylayed, Lacto-N-FucoPentaose I (LNFPI percentage), both di-fucosylated HMOs, an isomer of Lacto-N-difucosyl-hexaose (LNDFH with the following monosaccharides structure: 3210, i.e., 3 Hex/2 Fuc/1 HexNac/0 NeuAc) 4210d, and neutral HMOs such as pLNH (lacto-N-hexaose), but contained less neutral HMO 3000 and di-fucosylated HMO 4210b. Many of these HMOs were found to be variables of interest for breast milk glycome discrimination (VIP-PLS-DA index above 1.0, as reported in Table 4). Of note, breast milk presented no between-group differences in any sialylated HMOs. As reported in Table 4, many HMOs were significantly predictive of infant weight *Z*-score, as they successfully passed the multiple linear regression test (MLR *p*-value), following adjustment for maternal and infant confounding factors and after multiple correction testing (FDR-corrected MLR *q*-value significant, i.e., <0.1). Indeed, the fucosylated LNFP I, an isomer of LNDFH 3’FL (3’-fucosyllactose) and the neutral pLNH were predictive of infant weight Z-score for secretor mothers only, whereas, the four minor di-fucosylated HMOs (4230c, 4230b, 4240b, and 4210d) remained predictive of infant weight *Z*-score regardless of the secretor or non-secretor status of mothers.

### 3.5. Integration of Multi-Omics Data sets

**Figure 4:**
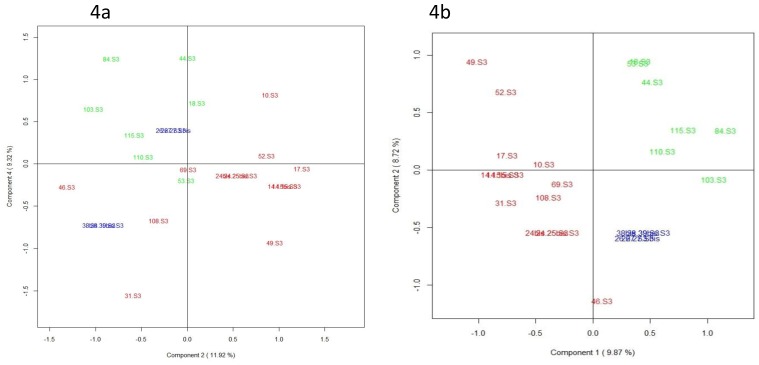
Score plot generated from UPCA (**a**) (with 21.4% of variance on components 3–4) and MB-PLS-DA (**b**) (with 18.5% of variance on components 1–2) based on the lipidomics/metabolomics/glycomics/FA/FAA profiles of human preterm milk at week 3 of lactation on the factor weight *Z*-score (discharge minus birth). Breast milk provided to preterm infants who experienced “faster” (green) or “slower” (red) growth and to twin infants with discordant growth rate, one twin with high growth rate and the other one with low growth rate (blue).

The aim of the current pilot study was to determine whether multi-block modeling could be applied for relating MS-based metabolomic, lipidomic, glycomic, fatty acids, and free amino acids data with regard to the predictive component that is the infant growth trajectory. Due to the complexity of longitudinal study by extracting the relevant information from multiple “omics” data sources, we chose to focus on “omics” data obtained on one representative time of lactation (week three of lactation). More specifically, the horizontal concatenation of annotated VIP provided by the AoV-PLS-DA model applied on MS-based metabolomic (i.e., 68 metabolites), lipidomic (i.e., 143 lipid species previously selected in [20]), and glycomic (79 HMOs) data with all metabolites–species issues from free amino acid and total fatty acid quantification was a straightforward solution to providing an extended analytical coverage of the biochemical diversity characterizing the breast milk samples. Concerning the glycomics data set, we had to overcome the problem due to the breast milk clustering based on maternal secretor or non-secretor status. As the relatively substantial variation in HMOs between the high and low 2’FL levels clusters were recently reported not to impact term infant growth of either sex up to four months [54], we chose to perform, on glycomic data, a mean-centered scaling of HMOs abundances on maternal secretor or non-secretor status before the “omics” data fusion. Then, on the super-matrix thus obtained, we tested UPCA or MBPLS multi-block strategies. Following the fusion of 340 selected annotated variables resulting from the combination of five data sources (blocks), the unsupervised UPCA score plot (Figure 4a) showed—on the principal components PC 3–4 that reported 21.4% variance—a clustering of breast milk samples and indicated a specific metabotype in the milk provided to preterm infants with “faster” growth versus that fed to infants with “slower” growth. The supervised MB-PLS score plot (Figure 4b) clearly highlighted on components 1–2 (18.5% of variance for the five blocks data), two breast milk metabotypes associated with ‘faster’ or ‘slower’ infant growth. The breast milk profiles, corresponding to the four sets of twins and depicted with blue symbols in Figure 4 were plotted between both “faster” and “slower” clusters. The most discriminant features associated with infant growth during hospital stay corresponded to a cluster of 87 metabolites species selected according to their loadings on the first MB-PLS components PC1 and PC2, and including: (i) many HMOs, such as LNFPI and 4210d, positively correlated with PC1 (i.e., associated with “faster” growth) or 4230b and 4230c, negatively correlated with PC1 (i.e., associated with “slower” growth), (ii) a few free amino acids (such as valine and glycine associated with “slower” growth), (iii) several lipidomic-species that were associated with “faster” growth, such as medium-chain saturated fatty acids (MCSAT, e.g., pentadecanoic and myristic acid), triglycerides (TG(46:0) and TG(50:2)), phospholipids (PS(38:4) and PE(38:3)), or that were associated with “slower” growth, such as oleic acid, plasmalogen-derivatives (PC(P-34:2) and PE(P-36:0)), lyso-phosphatidylethanolamine-containing arachidonic acid (LysoPE(20:4)), ceramide (Cer(18:1/22:0)), and very long-chain TG (TG(54:4) and TG(58:7)) and finally, (iv) few metabolomic-species positively (3-hydroxycapric acid, dihydrocaffeic acid 3-O-glucuronide, LNFPII) or negatively (9-undecenal, heptanoyl- and hexanoyl-glycine, 3-hydroxy-adipic acid, valerenic acid) correlated to PC1. Among these metabolites, MCSAT and oleic acid were previously shown to be predictive of optimal early growth [20].

## 4. Discussion

To date, only a few metabolomics studies have been reported on preterm human milk in the first few weeks of lactation [27,28]. To the best of our knowledge, the current pilot study is the first to comprehensively characterize and compare the preterm human milk lipidome (previously reported in [20]), metabolome, glycome, total fatty acid, and free amino acid profiles in relation to the growth velocity of preterm infant early in life in a group of 26 breastfeeding mother infant dyads. Our findings strongly suggest that molecular species other than “classic” macronutrients in human breast milk might affect infant growth early in life.

### 4.1. Higher Breast Milk Content in Branched-Chain and Insulino-Trophic Amino Acid and in Tyrosine Associated to Optimal Infant Growth

The higher levels of total branched-chain amino acids (BCAA) in breast milk provided to infants who experienced a “faster” early growth was consistent with the muscle protein anabolic effects of BCAA reported in adult humans [55]. In a previous study conducted on obese mothers of full-term infants, we found a 20% higher BCAA content in the breast milk obtained from obese mothers compared with control, lean mothers [56]. Several authors argued that the higher BCAA content of most formulas compared to human milk may contribute to the higher early weight gain observed in bottle-fed (compared to breastfed) full-term infants [57,58]. However, whether a high milk BCAA content directly impacts the growth of the breast-fed child remains to be explored. Among the insulino-trophic amino acid, arginine, the sole endogenous source of nitric oxide (NO), and a precursor of polyamines and creatine may regulate angiogenesis, mammary gland development, enhance protein synthesis, and decrease protein degradation in mammary epithelial cells [59], thereby improving lactation performance [60,61]. In our study, the optimal early infant growth associated with higher breast milk contents of arginine and carbamoylsarcosine, an intermediate in the creatine-arginine pathway, might reflect the positive effects of arginine and polyamine on muscle protein synthesis and on immune response [61], its beneficial effects on the intestinal mucosa itself, the prevention of necrotizing enterocolitis [62,63,64] and its protective effect for the nervous system [61]. Higher availability of creatine may serve as a source for phosphorylation in muscle tissue and fat metabolism. The higher amounts of tyrosine combined with lower abundances of tyrosine catabolism products (hydroxyphenylacetic acid and cresol) [23] observed in the “faster” breast milk are consistent with the key role of tyrosine in infant growth as a precursor of thyroxine, a hormone involved in energy metabolism, and of dopamine, a neurotransmitter.

### 4.2. Enhanced Milk Fat Availability by Infants with an Early “Faster” Growth Velocity

Our data highlighted lower concentrations of two free amino acids involved in bile acid conjugation, glycine and taurine, in the “faster” growth group. These data were observed along with an enhanced total lipid content previously reported in “faster” milk [20]. We speculate that the lower milk content in glycin and taurine, under their free form, along with the higher fat content could be explained by enhanced bile acid conjugation in “faster” breast milk, which could, consequently, facilitate the solubilization of lipids, sterols, and fat-soluble vitamins by forming mixed micelles, and, in turn, the uptake of these nutrients into enterocytes despite gastrointestinal immaturity. This also is in agreement with higher coefficients of fat absorption reported in breast-fed vs. formula-fed new borns, and related to the bile salt-dependent lipase (BSL) present in human milk [65]. The “faster” breast milk also presented decreased glycine derivatives, including hexanoyl- and heptanoylglycine, which are products of the mitochondrial fatty acid beta-oxidation [66]. These findings suggest low rates of fatty acids utilization in mammary gland and/or breast milk, which could enhance fat availability for breast-fed preterm infants. Moreover, the “faster” breast milk contained higher amounts in beta-hydroxybutyrate, a ketone body involved in the mammary gland synthesis of triglycerides, leading to the formation of milk fat globules [34]. Apart from its pivotal role as an energy fuel for extrahepatic tissues like brain, heart, or skeletal muscle in newborns, hydroxybutyrate is reported to play a role as a signaling mediator, a driver of protein post-translational modification, and a modulator of inflammation and oxidative stress [67]; such mechanisms could explain the link between beta-hydroxybutyrate availability and optimal early growth in a context of prematurity. Moreover, preterm breast milk metabolome showed decreased levels of few odd medium unbranched- (heptanoic and undecenal) or branched-chain (BCFAs, as 2-benzyloctanoic acid and methyl-2-octynoic acids) fatty acids but enhanced levels in one specific BCFA, the citraconic acid (known as methylmaleate, an odd and methyl-branched short chain unsaturated dicarboxylic fatty acid) in the “faster” group. Interestingly, mother’s milk BCFAs [68] have been reported to reduce the occurrence of necrotizing enterocolitis in a mouse model due to their active roles in reducing inflammation and altering microbiota in the gut [69]. However, the potential effects of BCFAs on the infant health and development remain to be examined [68], as Wongtangtintharn et al. [70] suggested that BCFA lowers fatty acid synthesis [71]. Indeed, the higher lipid content we previously reported in the “faster” breast milk lipidome [20] seems to be consitent with the lower levels of many BCFA observed in the present study. Additionally, the fact that odd chain saturated fatty acid are reported to pass into the milk of lactating cows [72] suggests that these fatty acids may also cross the blood-brain barrier and act on early postnatal brain development. Finally, the higher availability of niacinamide in “faster” milk suggests niacinamide could act, through its role as a precursor of co-enzymes NAD and NADP, and impact fatty acid utilization since NAD^+^ is used as a coenzyme in energy production (glycolysis, mitochondrial respiration) and improves gastrointestinal tract repair after damage as well as immune response [73].

Taken together, our findings suggest that the “faster” milk supplied (i) a higher content in energy, with calories supplied under a more digestible form of the preterm newborns, to overcome the immaturity of their digestive tractus, (ii) larger amounts or amino acids that promoted protein anabolism, and (iii) bioactive molecule, that played critical roles in energy homeostasis and gastrointestinal function, contributing to an optimal early-growth in infants born preterm. These findings also suggest that the higher lipid content in breast milk metabotype associated with preterm infants with optimal weight growth during hospitalization is used for enhanced fat oxidation rather than fat deposition, promoting tissue growth and likely a preferential fat-free mass deposition, which might in turn contribute to the recovery of the body composition and optimization of neurodevelopmental outcomes [74]. Moreover, the high BCAA and arginine intakes in breast milk provided to infants who belong to the “faster” group are also in agreement with this putative fat-free mass accretion in preterm infants, which could explain an optimal weight gain.

Additionnally, the higher content in phosphatidylcholine and sphingomyelin previously reported in the “faster” milk of the same pilot study [20] is consistent with the higher amounts of choline observed in breast milk metabolome provided to infants with optimal early-growth trajectory. Choline plays a key role as a precursor for acetylcholine (a neuromediator) and betaine (a source of labile methyl groups), which could increase the availability of methionine and choline and also enhance liver glycogenesis [75]. Indeed, this higher choline content may have a profound benefic impact upon the homestatic mechanisms and upon the physiological function in preterm infants, leading to an optimal growth during their hospitalization.

### 4.3. Di-Fucosylated HMOs Associated to Early Preterm Infant Growth

We found similar proportions of fucosylated and sialylated HMOs in preterm milk in good agreement with an earlier study from another team [31] but with slight differences in HMO-fucosylation level (according to the number of fucose residues carried) in both growth groups, such as a higher abundance of mono-(LNFPI) and a lower abundance of di- and tri-fucosylated oligosaccharides in breast milk provided to infants with “faster” growth. Given the small number of non-secretor mothers in this pilot study (five among 26 mothers) and because only one preterm milk sample was collected from a non-secretor mother who delivered a preterm infant with a “faster” growth, meaningful conclusions regarding differences in “faster” and “slower” growth milk on the basis of maternal secretor status cannot be drawn. There are scarce data to clarify the putative relationship between the maternal milk fucosyl-transferase 2 (FUT2)-dependent oligosaccharide status and infant anthropometry [32,54]. Recently, a specific FUT2-dependent HMO, LNFP I, but not 2’FL, was reported to be associated with the weight of full term infants at six months of age [32], but that association was not observed on growth curves over the first four months of life in groups of healthy, full term infants [54]. In our cohort, we observed consistent effects of the minor di-fucosylated HMOs (4230c, 4230b, 4240b, and 4210d) but not 2’FL and of the mono-fucosylated LNFP I (considering only secretors mother infant dyads) concentrations in breast milk on preterm infant growth up to 4 weeks of lactation. After multiple linear regression combined with multiple corrections and adjustment for confounding maternal and infant factors, the ability of the latter HMOs to predict early weight gain persisted. These di-fucosylated HMOs were also selected as discriminant metabolites in our MB-PLS model built on the five blocks of milk components. However, given the small sample size included in this exploratory study, future work would be needed in larger samples with longer follow-up to identify the exact contribution of specific HMOs to preterm infant growth. The impact of HMOS may involve effects on intestinal epithelium and gut microbiome, as fucosylated HMOs (and particularly LNFPI) support increased *bifidobacteria*, which dominate the microbiota of breastfed infants [76] but also on immune development and/or protection from infection through systemic effects [32], thereby likely affecting infant growth and body composition.

## 5. Conclusions

Previous studies have shown breastfeeeding is associated with many health benefits in pre-term infants. However, whether such benefits are due to mother’s milk constituents, *per se*, remains unclear. Breastmilk may not always be adequate for pre-term infants with high nutrition density requirements, which may lead to insufficient weight gain. We believe this to be the first study to document the changes in the metabolomics/lipidomics/glycomics/amino acids profiles of pre-term human milk during the first month of lactation, related to preterm infant growth during hospital stays. We showed that specific differences in milk metabolites exist between breast milk provided to preterm infants with optimal or non-optimal early growth, and a set of few milk metabolites were identified as predictive of infant growth parameters in the present pilot study, pointing to the critical role of energy utilization, protein synthesis, oxidative status, gut epithelial cell maturity, or more indirectly, gut microbiome in the context of prematurity. In particular, our findings highlighted robust biomarkers, i.e., arginine, tyrosine, hydroxybutyrate, niacinamide, choline, and lacto-N-fucopentaose I, that displayed a good ability to predict weight gain during hospital stays. Moreover, this preterm milk metabolomic signature suggests that the optimal early growth trajectory during hospital stays of preterm infants could be combined with preferential fat utilization and fat-free mass accretion. A clear limitation of our pilot study is its small population sample size and slight differences in birth weight between the groups. Although it has long been known that infants born with a lower birth weight grow faster [36,37], matching groups for birth weight would have been nearly impossible. We therefore have to admit that it cannot be ascertained whether the difference in breastmilk composition is programmed by infant antenatal growth or, alternatively, is one among the many determinant factors of postnatal growth trajectory. This study is also limited by the statistical method we applied because the Mann-Whitney *U* test has a lower power than the parametric method. Before the metabolites identified here can be considered valid biomarkers, they need to be quantified in the entire preterm newborn LACTACOL cohort and, ideally, validated in other longitudinal birth cohorts. Nevertheless, this pioneer integrative analysis for human breast milk might open the way to novel strategies using human milk as a tool to improve the outcome of a frail population of newborn infants. Indeed, providing optimal individual nutrition remains a daunting challenge and the subject of heated debate between neonatalogists on the issue of “individualized” fortification of human milk to improve growth and long term outcomes for all preterm infants.

## Figures and Tables

**Table 1:** Maternal and preterm infants’ characteristics.

Characteristics	“Faster” Growth Rate	“Slower” Growth Rate	*p*-Value
Maternal characteristics	11	11	
Age (years)	29.00 ± 4.52 (25.00; 35.00)	30.00 ± 4.12 (26.00; 33.00)	0.908
BMI before gestation (kg/m^2^)	22.32 ± 5.26 (19.14; 28.91)	24.00 ± 5.11 (20.83; 30.80)	0.789
Infants characteristics at birth	11 (7 males and 4 females)	15 (10 males and 5 females)	
Neonatal Morbidity (number of events) *	0	0	
Gestational age (weeks)	31.00 ± 1.37 (30.0; 32.00)	30.00 ± 1.68 (29.00; 32.00)	0.288
Length of hospital stay (days)	51.50 ± 3.16 (37.25; 56.25)	49.50 ± 4.21 (36.75; 54.75)	0.849
Birth weight (kg)	1.200 ± 0.293 (1.020; 1.445)	1.605 ± 0.211 (1.465; 1.705)	**0.005**
Birth weight Z-score (SD)	−1.592 ± 0.958 (−2.079; −0.571)	0.564 ± 0.718 (−0.290; 0.842)	**0.000**
BMI at birth (kg/m^2^)	7.694 ± 1.573 (7.139; 9.884)	9.455 ± 0.857 (8.843; 9.900)	0.161
Discharge weight (kg)	2.340 ± 0.320 (2.029; 2.520)	2.565 ± 0.270 (2.355; 2.720)	**0.041**
Discharge weight *Z*-score (SD)	−1.878 ± 0.857 (−2.264; −1.127)	−1.142 ± 0.682 (−1.552; −0.953)	0.146
BMI at Discharge (kg/m^2^)	11.98 ± 0.485 (11.66; 12.28)	12.67 ± 0.955 (11.78; 13.36)	**0.047**
Difference between discharge and birth weight *Z*-score (SD)	−0.479 ± 0.189 (−0.668; −0.294)	−1.538 ± 0.417 (−1.953; −1.230)	**<0.001**

*: The development of comorbidities was clearly described in the same pilot study [20]. Values are medians and 25% and 75% percentiles. *P* values for comparison between “faster” and “slower” growth groups were derived using Mann-Whitney *U* test. Parameters in bold presented a significant *p*-value < 0.05.

**Table 2:** Concentration levels of free amino acids in breast milk provided to preterm infants with “faster” or “slower” growth during the W2 to W4 lactation period.

Free Amino Acids (µM)	W2 to W4 Median (25% and 75% Percentile)		Mann-Whitney *p*-Value from W2 to W4	FDR Corrected MW *q*-Value from W2 to W4	MLR *p*-Value From W2 to W4	FDR Corrected MLR *q*-Value from W2 to W4
	“Slower” Growth (*n* = 38)	“Faster” Growth (*n* = 29)				
**EAA**	234.4 (216.9–278.5)	277.1 (221.4–343.5)	0.0675 *^t^*	0.18	0.06 #	0.08 #
Arginine	11.02 (7.56–21.84)	18.34 (11.45–28.13)	0.0079 **	0.05 *	0.10 #	0.08 #
Isoleucine	10.12 (7.56–14.51)	10.70 (8.12–18.01)	0.2224	0.29	0.08 #	0.44
Leucine	30.23 (19.93–35.46)	32.25 (24.00–39.02)	0.2407	0.29	0.05 #	0.40
Proline	30.21 (26.56–37.13)	30.32 (26.16–38.80)	0.9425	0.49	0.85	0.83
Methionine	5.29 (3.11–8.35)	6.27 (3.88–8.90)	0.6038	0.43	0.44	0.74
Phenylalanine	12.43 (8.81–15.47)	12.42 (7.25–16.43)	0.9277	0.48	0.48	0.77
Threonine	86.71 (74.34–115.7)	78.62 (66.76–107.5)	0.3197	0.31	0.39	0.71
Tryptophan	2.48 (1.89–4.23)	2.73 (1.97–4.22)	0.7676	0.45	0.59	0.21
Valine	51.13 (38.90–55.06)	52.09 (37.75–56.30)	0.8034	0.45	0.41	0.71
**NEAA**	2610 (2146–3280)	2512 (1753–3182)	0.2592	0.32	0.78	0.25
Alanine	206.7 (186.9–332.4)	201.0 (166.5–254.3)	0.5466	0.43	0.78	0.83
Aspartic acid & asparagine	66.93 (39.53–90.83)	57.39 (29.92–80.49)	0.3382	0.31	0.86	0.83
Glutamine	455.9 (211.4–902.3)	375.0 (134.4–572.0)	0.0838 *^t^*	0.17	0.14	0.52
Glutamic acid	1319 (898.5–1449)	1220 (906.3–1480)	0.6567	0.43	0.27	0.62
Glx	1838 (1381–2259)	1754 (1177–2098)	0.0613 *^t^*	0.14	0.52	0.21
Glycine	89.25 (68.99–105.2)	69.60 (54.38–103.09)	0.0126 *	0.05 *	0.18	0.56
Serine	86.01 (68.78–116.2)	86.01 (63.81–112.3)	0.8034	0.46	0.23	0.59
Tyrosine	11.64 (6.70–15.56)	14.49 (11.47–21.00)	0.0349 *	0.10	0.11	0.49
Taurine	313.9 (275.5–428.2)	270.0 (174.0–313.2)	0.0031 **	0.03 *	0.14	0.51
BCAA	85.15 (71.48–93.3)	101.1 (84.99–121.5)	0.0075 **	0.04 *	0.06 #	0.08 #
Insulino-trophic amino acid	182.0 (166.6–219.2)	224.9 (175.7–275.0)	0.0427 *	0.10 *^t^*	0.07 #	0.08 #
SAA	327.9 (297.0–430.7)	227.9 (168.6–355.1)	0.0019 **	0.03 *	0.13	0.09 #

Values are medians (25% and 75% percentiles) from amino acid concentrations from week 2 to week 4 of lactation period. EAA: essential amino acids; NEAA: non-essential amino acids; FAA: free amino acid; Glx: glutamine + glutamic acid; BCAA: branched chain amino-acids (valine + leucine + isoleucine); Insulinotrophic and glycemic amino acids = valine + leucine + isoleucine + threonine + arginine. Sulfur amino acids (SAA): taurine and methionine. Variables were considered as significantly modified between the two groups of infants’ growth (Mann-Whitney *U* test) when their multiple comparisons adjusted *P*-values (i.e., False Discovery Rate (FDR) corrected-MW *q*-value) was < 0.05. *: MW *p*-value or FDR-corrected MW *q*-value < 0.05; **: MW *p*-value or FDR-corrected MW *q*-value < 0.01; *t*: MW *p*-value or FDR-corrected MW *q*-value < 0.1. Multiple linear regression (MLR) for infant weight *Z*-score (*p*-value) was also combined to FDR and predictive ability for infant weight growth was considered reliable when MLR *q*-value was < 0.1 (#).

**Table 3:** Abundance (10^6^) of annotated metabolites that discriminated metabotypes of breast milk provided to preterm infants with “faster” or “slower” growth during the W2 to W4 lactation period.

			Abundance Des Ions (10^6^)			
Metabolites (Annotation Level)	a, b, c	mz	Median (25% and 75% Percentile), W2 to W4			
			“Slower“ Growth (*n* = 38)	“Faster“ Growth (*n* = 29)	Mann-Whitney *p*-Value (FDR-Corrected MW *q*-Value in Exposant)	MLR *p*-Value (FDR-Corrected MLR *q*-Value in Exposant)
Amino acid						
Hippuric acid ^1^	a	180.0654 (M + H)^+^	1.36 (0.79–1.81)	1.36 (0.88–2.20)	0.86	0.33
2-hydroxyhippuric acid ^2^	a, b, c	194.0459 (M – H)^−^	0.07 (0.05–0.126)	0.08 (0.05–0.15)	0.25	0.04
Valine ^1^	a	118.0865 (M + H)^+^	2.81 (1.72–3.23)	2.67 (0.91–3.17)	0.79	0.17
Leucine ^1^	a, c	130.0872 (M – H)^−^	1.92 (1.45–2.87)	2.99 (1.61–4.66)	0.02 **	0.92
N-Carbamoylsarcosine ^2^	a, c	133.0609 (M + H)^+^	0.96 (0.66–1.45)	1.69 (1.18–2.38)	0.0003 **	0.96
Tryptophan metabolism						
Tryptophan ^1^	a, c	205.0970 (M + H)^+^	4.20 (3.59–4.77)	4.76 (3.51–7.95)	0.18	0.79
Kynurenine ^1^	a, c	192.0653 (M–NH3 + H)^+^	0.97 (0.64–1.55)	0.72 (0.59–0.93)	*0.06 ^t^*	0.80
1H-Indole-3-carboxaldehyde ^2^	a, b	146.0599 (M + H)^+^	2.18 (1.55–2.81)	2.26 (1.66–3.63)	0.27	0.98
Indole-3-ethanol ^2^	a	184.0732 (M + Na)^+^	9.66 (6.11–13.01)	9.59 (6.62–12.06)	0.82	0.14
3-Methylindole ^2^	a	132.0806 (M + H)^+^	0.43 (0.35–0.55)	0.47 (0.39–0.63)	0.10	0.70
Tyrosine metabolism						
hydroxyphenylacetic acid ^1^	a, c	151.0399 (M – H)^−^	0.32 (0.25–0.37)	0.30 (0.21–0.37)	0.61	0.08
p-Cresol (4-methylphenol) ^2^	a, b, c	107.0501 (M – H)^−^	1.84 (1.16–2.85)	1.37 (0.09–2.21)	0.04 *	0.20
p-Cresol sulfate ^2^	a	187.0070 (M – H)^−^	5.28 (4.14–9.27)	4.89 (2.67–6.79)	0.13 *^t^*	0.17
Sulphur metabolism						
Cystathionine ^2^	a	240.1015 (M + NH_4_)^+^	0.67 (0.51–0.80)	0.87 (0.66–1.35)	0.02 *	0.90
Methionin ^1^	a, c	150.0580 (M + H)^+^	1.82 (1.49–2.19)	1.81 (1.63–2.69)	0.36	0.89
Se-Adenosylselenohomocysteine ^2^	a	228.0314 (M + H + Na)^+^	0.22 (0.17–0.25)	0.19 (0.13–0.24)	0.15 *^t^*	*0.09*
S-Adenosylhomocysteine ^2^	a	365.1048 (M – H_2_O– H)^+^	6.53 (5.02–7.63)	4.48 (3.92–6.84)	0.007 *	0.20
Hydrogen sulfite ^2^	a, b, c	79.9573 (M – H)^−^	0.52 (0.38–0.85)	0.44 (0.31–0.67)	*0.07 ^t^*	0.23
Thiocyanic acid ^2^	a, c	150.0018 (M – H)^−^	0.75 (0.64–0.83)	0.57 (0.51–0.76)	0.02 *	0.69
Aromatic compound						
Benzoic acid ^1^	a	121.0294 (M – H)^−^	3.14 (2.27–4.18)	2.49 (2.09–2.95)	0.01 *	0.16
Hydroxyphenyllactic acid ^2^	a	241.0730 (M + Hac-H)^−^	0.14 (0.11–0.16)	0.11 (0.08–0.18)	0.17	0.96
Pyridines and Derivatives/Nucleosides						
Niacinamide ^1^	a	123.0554 (M + H)^+^	6.45 (5.27–10.82)	11.00 (6.17–13.84)	0.029 *	0.16
Energy metabolism						
Hydroxyhexanoylcarnitine ^2^	a	276.1803 (M + H)^+^	0.11 (0.04–0.22)	0.08 (0.05–0.21)	0.84	0.52
Oxoicosanoyl-CoA ^2^	a, b	547.2129 (M + H+ NH4)^+^	0.66 (0.53–0.89)	0.81 (0.63–1.20)	0.04 *	0.87
3-Hydroxypimelyl-CoA ^2^	a	943.2103 (M + NH4)^+^	1.77 (1.66–2.02)	1.79 (1.61–2.04)	0.98	0.91
Hexanoylglycine ^2^	a, b	174.1123 (M + H)^+^	0.20 (0.12–0.26)	0.13 (0.08–0.19)	0.02 *	0.15
Heptanoylglycine ^2^	a	229.1544 (M + H)^+^	0.64 (0.50–0.97)	0.65 (0.49–0.81)	0.58	0.69
Gamma-Butyrolactone/	a	85.0293 (M – H)^−^	42.9 (25.08–58.13)	48.37 (34.67–65.61)	0.32	0.68
But-2-enoic/Isocrotonic acid ^2^	a, b	631.3089 (M – H)^−^	0.01 (0.00–0.03)	0.02 (0.01–0.06)	0.01 *	0.63
butyl 2-dodecanoic acid/ 5-Tetra dodecanoic acid ^2^	a	225.1859 (M – H)^−^	0.09 (0.04–0.19)	0.06 (0.01–0.10)	*0.05 ^t^*	0.81
caproic acid ^1^	a	115.0763 (M – H)^−^	0.13 (0.11–0.15)	0.14 (0.11–0.18)	0.46	0.26
3-hydroxycapric acid ^2^	a, b, c	187.1339 (M – H)^−^	0.43 (0.26–0.81)	0.62 (0.50–1.11)	0.01 *	0.97
Geranic acid ^2^		167.1077 (M – H)^−^	0.09 (0.07–0.22)	0.08 (0.06–0.16)	0.13	0.03
Sebacic acid ^1^	a, c	261.1345 (M-CH3COO)^−^	0.10 (0.07–0.18)	0.13 (0.08–0.19)	0.18 *^t^*	0.72
3-Hydroxysebacic acid ^2^	a, c	217.1081 (M – H)^−^	0.04 (0.03–0.06)	0.06 (0.03–0.07)	*0.07 ^t^*	0.68
3,4-Methylenesebacic acid ^2^	a	225.1132 (M – H)^−^	0.04 (0.03–0.06)	0.03 (0.02–0.04)	0.12 *^t^*	0.82
2-Hydroxybutyric acid ^1^	a, b, c	103.0399 (M – H)^−^	2.93 (2.35–3.72)	3.85 (3.05–4.81)	0.005 *	0.77
2-hydroxy-3-methylbutyric acid ^1^	a, b, c	117.0555 (M – H)^−^	0.99 (0.81–1.82)	1.52 (1.07–2.07)	0.01 *	*0.06*
pyridosine ^2^	a, c	253.1195 (M – H)^−^	0.20 (0.09–0.28)	0.11 (0.07–0.22)	*0.06 ^t^*	0.17
Glycerophosphorylcholine ^2^	a, c	292.0724 (M – H)^−^	0.68 (0.49–0.96)	0.58 (0.41–0.71)	*0.07 ^t^*	0.40
N-Heptanoylglycine ^2^	a, b	186.1135 (M – H)^−^	0.93 (0.47–1.83)	0.61 (0.40–1.16)	*0.07 ^t^*	0.14
Butyryl glycine/Saccharopine ^2^	a	335.1455 * (M + Fa − H)^−^	0.22 (0.11–0.53)	0.28 (0.08–0.51)	0.58	0.62
2-Phenylglycine ^2^	a, c	150.0559 (M – H)^−^	0.14 (0.09–0.22)	0.17 (0.10–0.29)	0.11 *^t^*	*0.08*
Cis-aconitic acid ^1^	a	154.9983 (M-H_2_O – H) ^−^	1.33 (0.58–2.27)	1.73 (1.28–2.49)	*0.09 ^t^*	0.85
Pyruvic acid ^1^	a, b, c	147.0297 (M-CH3COO)^−^	2.15 (1.27–3.49)	3.55 (1.94–6.10)	0.03 *	0.95
Citraconic ^1^	a, b, c	129.0192 (M – H)^−^	13.82 (9.49–26.19)	21.43 (13.82–29.91)	0.02 *	0.95
2-Keto-glutaramic acid ^2^	a, c	144.0302 (M – H)^−^	0.38 (0.30–0.44)	0.37 (0.31–0.58)	0.21	0.44
Panthothenic acid ^1^	a, c	200.0929 (M-H_2_O – H)^−^	0.09 (0.06–0.11)	0.10 (0.08–0.11)	0.51	0.007
4-Heptenal ^2^	a, b	111.0814 (M – H)^−^	0.43 (0.33–0.50)	0.35 (0.26–0.45)	0.04 *	0.55
2-Methylpentanal ^2^	a	99.0814 (M – H)^−^	0.12 (0.09–0.13)	0.10 (0.09–0.12)	*0.06 ^t^*	0.98
Undecenal ^2^	a, b, c	167.1440 (M – H)^−^	0.08 (0.06–0.12)	0.06 (0.04–0.13)	0.006 *	0.02
Methyl 2-octynoate ^2^	a	153.0919 (M – H)^−^	0.10 (0.07–0.13)	0.07 (0.06–0.11)	*0.09 ^t^*	0.06
4-Methylphenyl-acetaldehyde ^2^	a	133.0658 (M – H)^−^	0.07 (0.05–0.11)	0.06 (0.05–0.09)	0.04 *	0.14
4-Hydroxynonenal ^2^	a, c	155.1077 (M – H)^−^	0.06 (0.04–0.09)	0.07 (0.05–0.10)	0.13 *^t^*	0.79
cis-4-Decenedioic acid ^2^	a, b, c	199.0973 (M – H)^−^	0.07 (0.05–0.11)	0.10 (0.07–0.16)	0.01 *	0.94
Tetradecanedioic acid ^1^	a, c	257.1761 (M – H)^−^	0.08 (0.05–0.12)	0.12 (0.08–0.19)	*0.07 ^t^*	0.62
Dodecanedioic acid ^2^	a, c	229.1445 (M – H)^−^	0.41 (0.30–0.56)	0.35 (0.30–0.54)	0.42	0.01
Heptanoic acid ^2^	a, c	129.0920 (M – H)^−^	0.17 (0.14–0.21)	0.14 (0.10–0.19)	*0.06 ^t^*	0.74
2-benzyloctanoic acid ^2^	a, b, c	233.1544 (M – H)^−^	1.45 (0.78–2.31)	0.87 (0.61–1.45)	0.007 *	0.81
N-methylethanolaminium phosphate ^2^	a, b, c	136.0165 (M–H_2_O – H)^−^	0.36 (0.27–0.51)	0.26 (0.19–0.30)	<0.0001 **	0.81
Phosphorylcholine ^1^	a,	206.0551 (M + Na)^+^	5.78 (4.26–6.63)	4.03 (0.68–5.67)	0.001 *	0.21
Glycerophosphocholin ^2^	a	280.0917 (M + Na)^+^	18.88 (9.66–30.18)	16.98 (3.54–24.57)	0.12 *^t^*	0.57
Choline ^1^	a,	105.11080 (M + H)^+^	2.67 (2.25–3.66)	3.30 (2.74–4.65)	0.008 *	0.02
Glucuronide/oligosides						
Dihydrocaffeic acid 3-O-glucuronide ^2^	a, b, c	383.0763 (M + Na)^+^	1.15 (0.87–1.29)	1.05 (0.86–1.23)	0.49	0.51
2-Fucosyllactose ^2^	a, c	511.1629 (M + H)^+^	106.6 (82.1–152.5)	122.6 (98.5–148.7)	0.34	0.79
N-acetyl-D-glucosamine ^2^	a	244.0788 (M + Na)^+^	2.94 (2.71–3.51)	3.20 (2.55–3.81)	0.74	0.68
Lacto-N-fucopentaose-2 ^2^	a, b	876.2936 (M + Na)^+^	9.29 (7.62–12.68)	10.56 (8.07–15.04)	0.15 *^t^*	0.72
Saccharopine ^2^	a, c	335.1455 (M-CH3COO)^−^	0.22 (0.11–0.53)	0.28 (0.08–0.51)	0.58	0.62

Values are medians (25% and 75% percentiles) from metabolites abundances from week 2 to week 4 of lactation period. Metabolites’ annotation level in brackets: 1: identification level, definitively annotated with our home data base (i.e., based upon characteristic physicochemical properties of chemical reference standards (m/z, RT) and their MS/MS spectra compared to those of breastmilk QC); 2: putatively annotated compounds (i.e., without chemical reference standards, based upon physicochemical properties and MS/MS spectral similarity with public/commercial spectral libraries). (**a**) VIP in AoV-PLS/LDA ESI^+^ or ESI^−^ model, (**b**) loadings in MB-PLS model, (**c**) loadings in ACC model; when the letters (a, b or c) are in italic, that means that the significance of metabolites, as VIP or loadings in statistical models, is just a trend. Variables were considered as significantly modified between the two groups of infants’ growth (Mann-Whitney U test) when their multiple comparisons adjusted *P*-values (i.e., FDR corrected-MW *q*-value) was < 0.05. FDR-corrected MW *q*-value was labelled in exposant: *: FDR-corrected *q*-value < 0.05; **: FDR-corrected MW *q*-value < 0.01; t: FDR-corrected MW *q*-value < 0.1. Predictive ability of metabolites for infant weight growth was considered reliable when FDR-corrected MLR *q*-value was < 0.1 (and labelled in exposant as #).

**Table 4:** Major HMOs detected in breast milk glycome and provided to preterm infants with “faster” or “slower” growth during the W2 to W4 lactation period.

HMOs	Composition						Median (25% and 75% Percentile) from W2 to W4 (Secretors and Non Secretors Mothers)		Mann-Whitney *p*-Value (FDR-Corrected MW *q*-Value in Exposant)		VIP –PLS-DA (C1–C2)		MLR-*p*-value (FDR-Corrected MLR *q*-value in Exposant)	
	mz	RT	Hex	Fuc	HexNac	NeuAc	“Slower” Growth (*n* = 38)	“Faster” Growth (*n* = 29)	Secretors and Non Secretors	Secretors Only	Secretors and Non Secretors	Secretors Only	Secretors and Non Secretors	Secretors Only
Fucosylated							61.46 (50.28–65.13)	62.82 (60.20–65.19)	0.1847 *^t^*	0.1370				0.73
Sialylated							8.47 (6.97–9.40)	7.45 (6.70–9.11)	0.2545	0.2773				0.37
Fucosylated./Sialylated							1.95 (1.50-2.32)	1.63 (1.33–2.30)	*0.0973 **^t^***	0.1422				0.35
Neutral							28.30 (26.78–38.23)	26.87 (25.92–29.57)	0.0352 *	0.6966				0.95
Mono Fucosylated							28.49 (19.06–34.74)	36.95 (31.83-39.21)	0.0020	0.0669				0.19
Di Fucosylated							26.64 (23.83–28.53)	23.12 (19.14–26.77)	0.0026	0.0054 *				0.10
Tri Fucosylated							3.52 (2.96–4.00)	2.85 (2.63–3.26)	0.0061	*0.0883*				0.43
Tetra Fucosylated							3.20 (2.54–4.07)	2.94 (1.77–3.83)	0.1520	0.0243 *				0.004 *
LNFPI≠	856.3280	10.2	3	1	1	0	11.99 (0.36-18.65)	19.86 (15.00–23.69)	0.0003 **	0.0045 *	1.22	1.26	0.63	0.03 *
pLNH≠	1075.4023	18	4	0	2	0	0.26 (0.14–0.35)	0.38 (0.26–0.54)	0.0014 **	0.0013 *	1.44	1.75	0.50	0.10 *
2’-FL≠	491.1958	8.5	2	1	0	0	11.22 (0.10–13.87)	12.58 (10.29–15.09)	*0.0882 ^t^*	0.9433	1.10	1.08	0.63	0.22 *
6’-SL	636.2333	9.6	2	0	0	1	2.50 (2.30–3.06)	2.47 (1.81–3.05]	0.3219	0.9433	0.65	0.29	0.26	0.31 *
LNnH≠	1075.4023	15.1	4	0	2	0	0.82 (0.32–1.44)	0.95 (0.58–2.31)	*0.0689 ^t^*	0.8116	1.25	1.22	0.67	0.52 *^t^*
LNT/LNnT≠	710.2701	10.9	3	0	1	0	22.52 (20.29–35.65)	22.46 (20.04–25.21)	0.1991 *^t^*	0.2591	1.00	0.81	0.48	0.62 *^t^*
LSTc/b	1001.3655	18.4	3	0	1	1	2.59 (2.00–3.57)	2.63 (2.25–3.41)	0.7012	0.1422	0.89	0.87	0.78	0.65 *^t^*
LNDFH I	1002.3859	5.7	3	2	1	0	4.26 (0.44–6.25)	5.10 (0.51–6.96)	0.3986	0.8903	0.55	0.74	0.22	0.50 *^t^*
3’ FL	491.1958	1.8	2	1	0	0	0.03 (0.00–0.08)	0.00 (0.00–0.03)	0.0133 *	0.1697	0.94	0.70	0.12	0.09 *
3’SL	636.2333	18.9	2	0	0	1	0.15 (0.13–0.21)	0.14 (0.11–0.18)	0.1555 *^t^*	0.9433	0.46	0.48	0.26	0.16 *
LNDFH ≠	1002.3859	9.6	3	2	1	0	0.26 (0.14-0.36)	0.36 (0.27-0.45)	0.0020 **	0.1050	1.12	1.05	0.43	0.04 *
LNDFHx	1002.3859	6.8	3	2	1	0	0.26 (0.11–1.11)	0.14 (0.07–0.24)	0.0293 *	*0.0726 ^t^*	0.97	1.04	0.15	0.06 *
4230c≠	1513.5760	13.5	4	2	3	0	0.11 (0.00–0.18)	0.07 (0.04–0.14)	0.5405	0.0002 **	1.90	1.55	0.0062 *	0.07 *
4210d≠	1221.4602	17.4	4	2	1	0	0.18 (0.00–0.39)	0.47 (0.27–1.31)	0.0005 **	0.0142 *	1.69	1.66	0.10	0.04 *
4220e≠	1367.5181	13	4	2	2	0	1.59 (0.37–2.43)	1.75 (1.35–2.77)	0.1799 *^t^*	0.6128	1.28	1.76	0.86	0.78 *^t^*
4230b≠	1513.576	8.2	4	2	3	0	0.65 (0.00–1.49)	0.61 (0.06–1.20)	0.7967	*0.0893 ^t^*	1.38	1.33	0.0095 *	0.09 *
3000≠	507.1907	6.4	3	0	0	0	0.26 (0.19–0.31)	0.16 (0.12–0.23)	<0.0001 ***	0.0022 *	2.06	0.90	0.53	0.22 *
5300 (2+)	720.7709	18.1	5	3	0	0	0.15 (0.10–0.23)	0.16 (0.14–0.32)	0.0771 *^t^*	0.6305	1.41	1.39	0.72	0.37 *
6420c (2+)≠	1049.3949	18.1	6	4	2	0	0.44 (0.09–0.64)	0.50 (0.32–0.72)	0.1893 *^t^*	0.5220	1.11	0.67	0.30	0.76 *^t^*
6430d (2+)≠	1122.4238	16.8	6	4	3	0	0.14 (0.00–0.26)	0.15 (0.10–0.24)	0.2102 *^t^*	0.2663	1.18	1.00	0.34	0.87 *^t^*
5310c	1586.5924	18	5	3	1	0	0.29 (0.22–0.38)	0.38 (0.25–0.46)	0.0428 *	0.4094	0.90	1.02	0.95	0.16 *
2110a	694.2752	3.6	2	1	1	0	0.01 (0.00–0.27)	0.24 (0.00–0.51)	0.0811 *^t^*	0.6772	0.90	1.49	0.48	0.60 *^t^*
4240b≠	1659.6339	13.9	4	2	4	0	0.04 (0.00–0.11)	0.04 (0.00–0.08)	0.9654	0.0457 *	1.29	0.68	0.0007 **	0.05 *
2020a≠	637.2537	11.1	2	0	2	0	0.49 (0.04–0.61)	0.55 (0.45–0.66)	0.0840 *^t^*	0.9700	1.10	1.23	0.72	0.20 *
5310b	1586.5924	17	5	3	1	0	0.24 (0.13–0.31)	0.16 (0.09–0.27)	0.1091 *^t^*	0.0420 *	0.93	0.97	0.84	0.40 *
4220a	684.2627	7.7	4	2	2	0	0.20 (0.14–0.29)	0.11 (0.07–0.25)	0.0216 *	*0.0911 ^t^*	0.49	0.52	0.10	0.82 *^t^*
4220b≠	1367.5181	8.8	4	2	2	0	0.31 (0.09–1.78)	0.23 (0.07–0.33)	0.0895 *^t^*	0.3352	1.02	0.99	0.15	0.14 *
4210b≠	1221.4602	12.6	4	2	1	0	7.19 (5.03–8.90)	3.75 (2.37–6.33)	<0.0001 ***	0.0003 **	1.05	1.84	0.30	0.07 *
5330b	1878.7082	13.9	5	3	3	0	0.23 (0.00–0.39)	0.29 (0.00–0.38)	0.3749	0.4106	0.96	1.10	0.05	0.26 *
5320a	1732.6503	12.9	5	3	2	0	0.27 (0.13–0.70)	0.13 (0.05–0.22)	0.0004 **	0.0045 *	1.06	1.37	0.47	0.16 *

Relative HMO abundances (%) were calculated by dividing absolute HMO peak area by each sample’s total HMO peak areas. Values are medians (25% and 75% percentiles) from relative HMOs abundances from week 2 to week 4 of lactation period. P values for comparison between “faster” and “slower” growth groups were derived using Mann-Whitney *U* test. Variables were considered as significantly modified between the two groups of infants’ growth when their multiple comparisons adjusted *P*-values (e.g., q-value after false discovery rate) was < 0.05. *: FDR-corrected MW *q*-value < 0.05; **: FDR-corrected MW *q*-value < 0.01; ***: FDR-corrected MW *q*-value < 0.001; *t*: FDR-corrected MW *q*-value < 0.1. Multiple Linear Regression (MLR) for infant weight Z-score (p-value) was also combined with FDR, and predictive ability for infant weight growth was considered reliable when MLR *q*-value was < 0.05. *: FDR-corrected MLR *q*-value < 0.05; *t*: FDR-corrected MLR *q*-value < 0.1. ≠: variables of importance for PLS-DA model (VIP > 1.0). LNDFH, Lacto-N-difucosyl-hexaose; LNT, lacto-N-tetraose; pLNH, p-Lacto-N-Hexaose; LNFP, lacto-N-fucopentaose; LNnT, lacto-N-neotetraose; LNT, lactoN-tetraose; LST, sialyl-lacto-N-tetraose; 2’-FL, 2’-fucosyllactose; 3-FL, 3-fucosyllactose; 3’-SL, 3’-sialyllactose; Hex, hexose; HexNac, N-acetylhexosamine; Fuc, fucose and NeuAc, N-acetylneuraminic acid. Fucosylation was further investigated to determine the differences in the abundance of mono, di, tri, and tetrafucosylation (based on the number of fucose residues).

## Supplementary Materials

The following are available online at https://www.mdpi.com/2072-6643/11/3/528/s1. Figure S1: AoV-PLS and LDA models, based on the LC-ESI--HRMS metabolomics profiles of human preterm milk, on the factor weight *Z*-score (discharge-birth): AoV-PLS score plot with 63% of variance (R2Y = 218%) on components 1–2 (Figure S1a) and LDA (built on components of AoV-PLS) with a *p*-value = 0) (Figure S1b). Breast milk provided to preterm infants who experienced ‘faster’ (green) or ‘slower’ (red) growth and to twin infants with discordant growth rate, one twin with high growth rate and the other one with low growth rate, (blue).

## Abbreviations

AoV-PLS	analysis of variance combined to partial least squares regression
BCFA	branched-chain fatty acids
ESI	electrospray ionization
FDR	false discovery rate
GA	gestational age
HMO	human milk oligosaccharides
LC-HR-MS	Liquid-Chromatography-High-Resolution-Mass-Spectrometry
MG PLS-DA	multi-group partial least squares discriminant analysis
MLR	multiple linear regression
MCSAT	medium-chain saturated fatty acid
U-PCA	unfold principal component analysis
SD	standard deviation
W4M	Workflow4Metabolomics^®^
