# Correction: Alexandre-Gouabau et al. “Comprehensive Preterm Breast Milk Metabotype Associated with Optimal Infant Early Growth Pattern”, *Nutrients*, 2019, *11*, 528

**DOI:** 10.3390/nu12010162

**Published:** 2020-01-07

**Authors:** Marie-Cécile Alexandre-Gouabau, Thomas Moyon, Agnès David-Sochard, François Fenaille, Sophie Cholet, Anne-Lise Royer, Yann Guitton, Hélène Billard, Dominique Darmaun, Jean-Christophe Rozé, Clair-Yves Boquien

**Affiliations:** 1 INRA, UMR1280, Physiopathologie des Adaptations Nutritionnelles, Institut des maladies de l’appareil digestif (IMAD), Centre de Recherche en Nutrition Humaine Ouest (CRNH), F-44093 Nantes, France; Thomas.Moyon@univ-nantes.fr (T.M.); agnes.david@univ-nantes.fr (A.D.-S.); helene.billard@univ-nantes.fr (H.B.); dominique.darmaun@chu-nantes.fr (D.D.); jeanchristophe.roze@chu-nantes.fr (J.-C.R.); clair-yves.boquien@univ-nantes.fr (C.-Y.B.); 2 Service de Pharmacologie et d’Immunoanalyse, Laboratoire d’Etude du Métabolisme des Médicaments, CEA, INRA, Université Paris Saclay, MetaboHUB, F-91191 Gif-sur-Yvette, France; francois.fenaille@cea.fr (F.F.); sophie.cholet@cea.fr (S.C.); 3 LUNAM Université, ONIRIS, Laboratoire d’Etude des Résidus et Contaminants dans les Aliments (LABERCA), USC INRA 1329, F-44307 Nantes, France; anne-lise.royer@oniris-nantes.fr (A.-L.R.); yann.guitton@oniris-nantes.fr (Y.G.); 4 CHU, Centre Hospitalo-Universitaire Hôtel-Dieu, F-44093 Nantes, France; 5 EMBA, European Milk Bank Association, I-20126 Milano, Italy

The authors wish to make a correction to Section 2.7 and Table 4 in the published version of their paper [1].

A reverse digit error was found when reporting the monosaccharide compositions of Human Milk Oligosaccharides (HMOs) in Table 4 and, in particular, in the order of hexose (Hex), N-acetylhexosamine (HexNac), fucose (Fuc), and N-acetylneuraminic acid (NeuAc) numbers in the first 12 HMOs. A corrected version of Table 4 is shown below.

One edit to the sentence in the paragraph of *2.7. Lipidomic, Metabolomic, and Glycomic Data Treatment and Metabolites Annotation* is needed: “When it was not possible to clearly determine HMO structures, HMOs were named according to their monosaccharide compositions and denoted as hexose (Hex), fucose Fuc), N-acetylhexosamine (HexNac), and N-acetylneuraminicacid (NeuAc) numbers”. This sentence should now read: “When it was not possible to clearly determine HMOs structures, HMOs were named according to their monosaccharide compositions and denoted as hexose (Hex), N-acetylhexosamine (HexNAc), fucose (Fuc), and N-acetylneuraminic acid (NeuAc) numbers”.

This change does not affect anything in the overall results of the selection of HMO biomarkers of infant early growth.

The authors would like to apologize for any inconvenience caused to readers by these changes.

## Figures and Tables

**Table 4:** Major Human Milk Oligosaccharides HMOs detected in breast milk glycome and provided to preterm infants with “faster” or “slower” growth during the W2 to W4 lactation period.

HMOs	Composition						Median (25% and 75% Percentile) from W2 to W4 (Secretors and Non-Secretor Mothers)		Mann–Whitney *p*-value (FDR-Corrected MW *q*-Value in Exposant)		VIP–PLS-DA (C1–C2)		MLR *p*-Value (FDR-Corrected MLR *q*-Value in Exposant)	
	mz	RT	Hex	HexNAc	Fuc	NeuAc	“Slower” Growth (*n* = 38)	“Faster” Growth (*n* = 29)	Secretors and Non-Secretors	Secretors Only	Secretors and Non-Secretors	Secretors Only	Secretors and Non-Secretors	Secretors Only
Fucosylated							61.46 (50.28–65.13)	62.82 (60.20–65.19)	0.1847 *^t^*	0.1370				0.73
Sialylated							8.47 (6.97–9.40)	7.45 (6.70–9.11)	0.2545	0.2773				0.37
Fucosylated./Sialylated							1.95 (1.50–2.32)	1.63 (1.33–2.30)	0.0973 ^t^	0.1422				0.35
Neutral							28.30 (26.78–38.23)	26.87 (25.92–29.57)	0.0352 *	0.6966				0.95
Mono Fucosylated							28.49 (19.06–34.74)	36.95 (31.83–39.21)	0.0020	0.0669				0.19
Di Fucosylated							26.64 (23.83–28.53)	23.12 (19.14–26.77)	0.0026	0.0054 *				0.10
Tri Fucosylated							3.52 (2.96–4.00)	2.85 (2.63–3.26)	0.0061	0.0883				0.43
Tetra Fucosylated							3.20 (2.54–4.07)	2.94 (1.77–3.83)	0.1520	0.0243 *				0.004 *
LNFPI≠	856.3280	10.2	3	1	1	0	11.99 (0.36–18.65)	19.86 (15.00–23.69)	0.0003 **	0.0045 *	1.22	1.26	0.63	0.03 *
pLNH≠	1075.4023	18	4	2	0	0	0.26 (0.14–0.35)	0.38 (0.26–0.54)	0.0014 **	0.0013 *	1.44	1.75	0.50	0.10 *
2′-FL≠	491.1958	8.5	2	0	1	0	11.22 (0.10–13.87)	12.58 (10.29–15.09)	0.0882 *^t^*	0.9433	1.10	1.08	0.63	0.22 *
6′-SL	636.2333	9.6	2	0	0	1	2.50 (2.30–3.06)	2.47 (1.81–3.05]	0.3219	0.9433	0.65	0.29	0.26	0.31 *
LNnH≠	1075.4023	15.1	4	2	0	0	0.82 (0.32–1.44)	0.95 (0.58–2.31)	0.0689 *^t^*	0.8116	1.25	1.22	0.67	0.52 *^t^*
LNT/LNnT≠	710.2701	10.9	3	1	0	0	22.52 (20.29–35.65)	22.46 (20.04–25.21)	0.1991 *^t^*	0.2591	1.00	0.81	0.48	0.62 *^t^*
LSTc/b	1001.3655	18.4	3	1	0	1	2.59 (2.00–3.57)	2.63 (2.25–3.41)	0.7012	0.1422	0.89	0.87	0.78	0.65 *^t^*
LNDFH I	1002.3859	5.7	3	1	2	0	4.26 (0.44–6.25)	5.10 (0.51–6.96)	0.3986	0.8903	0.55	0.74	0.22	0.50 *^t^*
3′ FL	491.1958	1.8	2	0	1	0	0.03 (0.00–0.08)	0.00 (0.00–0.03)	0.0133 *	0.1697	0.94	0.70	0.12	0.09 *
3′ SL	636.2333	18.9	2	0	0	1	0.15 (0.13–0.21)	0.14 (0.11–0.18)	0.1555 *^t^*	0.9433	0.46	0.48	0.26	0.16 *
LNDFH ≠	1002.3859	9.6	3	1	2	0	0.26 (0.14-0.36)	0.36 (0.27-0.45)	0.0020 **	0.1050	1.12	1.05	0.43	0.04 *
LNDFHx	1002.3859	6.8	3	1	2	0	0.26 (0.11–1.11)	0.14 (0.07–0.24)	0.0293 *	*0.0726 ^t^*	0.97	1.04	0.15	0.06 *
4230c≠	1513.5760	13.5	4	2	3	0	0.11 (0.00–0.18)	0.07 (0.04–0.14)	0.5405	0.0002 **	1.90	1.55	0.0062 *	0.07 *
4210d≠	1221.4602	17.4	4	2	1	0	0.18 (0.00–0.39)	0.47 (0.27–1.31)	0.0005 **	0.0142 *	1.69	1.66	0.10	0.04 *
4220e≠	1367.5181	13	4	2	2	0	1.59 (0.37–2.43)	1.75 (1.35–2.77)	0.1799 *^t^*	0.6128	1.28	1.76	0.86	0.78 *^t^*
4230b≠	1513.576	8.2	4	2	3	0	0.65 (0.00–1.49)	0.61 (0.06–1.20)	0.7967	*0.0893 ^t^*	1.38	1.33	0.0095 *	0.09 *
3000≠	507.1907	6.4	3	0	0	0	0.26 (0.19–0.31)	0.16 (0.12–0.23)	<0.0001 ***	0.0022 *	2.06	0.90	0.53	0.22 *
5300 (2+)	720.7709	18.1	5	3	0	0	0.15 (0.10–0.23)	0.16 (0.14–0.32)	0.0771 *^t^*	0.6305	1.41	1.39	0.72	0.37 *
6420c (2+)≠	1049.3949	18.1	6	4	2	0	0.44 (0.09–0.64)	0.50 (0.32–0.72)	0.1893 *^t^*	0.5220	1.11	0.67	0.30	0.76 *^t^*
6430d (2+)≠	1122.4238	16.8	6	4	3	0	0.14 (0.00–0.26)	0.15 (0.10–0.24)	0.2102 *^t^*	0.2663	1.18	1.00	0.34	0.87 *^t^*
5310c	1586.5924	18	5	3	1	0	0.29 (0.22–0.38)	0.38 (0.25–0.46)	0.0428 *	0.4094	0.90	1.02	0.95	0.16 *
2110a	694.2752	3.6	2	1	1	0	0.01 (0.00–0.27)	0.24 (0.00–0.51)	0.0811 *^t^*	0.6772	0.90	1.49	0.48	0.60 *^t^*
4240b≠	1659.6339	13.9	4	2	4	0	0.04 (0.00–0.11)	0.04 (0.00–0.08)	0.9654	0.0457 *	1.29	0.68	0.0007 **	0.05 *
2020a≠	637.2537	11.1	2	0	2	0	0.49 (0.04–0.61)	0.55 (0.45–0.66)	0.0840 *^t^*	0.9700	1.10	1.23	0.72	0.20 *
5310b	1586.5924	17	5	3	1	0	0.24 (0.13–0.31)	0.16 (0.09–0.27)	0.1091 *^t^*	0.0420 *	0.93	0.97	0.84	0.40 *
4220a	684.2627	7.7	4	2	2	0	0.20 (0.14–0.29)	0.11 (0.07–0.25)	0.0216 *	0.0911 *^t^*	0.49	0.52	0.10	0.82 *^t^*
4220b≠	1367.5181	8.8	4	2	2	0	0.31 (0.09–1.78)	0.23 (0.07–0.33)	*0.0895 ^t^*	0.3352	1.02	0.99	0.15	0.14 *
4210b≠	1221.4602	12.6	4	2	1	0	7.19 (5.03–8.90)	3.75 (2.37–6.33)	<0.0001 ***	0.0003 **	1.05	1.84	0.30	0.07 *
5330b	1878.7082	13.9	5	3	3	0	0.23 (0.00–0.39)	0.29 (0.00–0.38)	0.3749	0.4106	0.96	1.10	0.05	0.26 *
5320a	1732.6503	12.9	5	3	2	0	0.27 (0.13–0.70)	0.13 (0.05–0.22)	0.0004 **	0.0045 *	1.06	1.37	0.47	0.16 *

Relative HMO abundances (%) were calculated by dividing absolute HMO peak area by each sample’s total HMO peak areas. Values are medians (25% and 75% percentiles) from relative HMOs abundances from week 2 to week 4 of lactation period. *P*-values for comparison between “faster” and “slower” growth groups were derived by using Mann–Whitney *U* test. Variables were considered significantly modified between the two groups of infants’ growth when their multiple comparisons adjusted *p*-values (e.g., *q*-value after False Discovery Rate (FDR)) was <0.05. *: FDR-corrected MW *q*-value < 0.05; **: FDR-corrected MW *q*-value < 0.01; ***: FDR-corrected MW *q*-value < 0.001; *t*: FDR-corrected MW *q*-value < 0.1. Multiple Linear Regression (MLR) for infant weight *Z*-score (*p*-value) was also combined with FDR and predictive ability for infant weight growth were considered reliable when MLR *q*-value was <0.05. *: FDR-corrected MLR *q*-value < 0.05; *t*: FDR-corrected MLR *q*-value < 0.1. ≠: variables of importance for Partial Least Square-Discriminant Analysis (PLS-DA) model (VIP > 1.0). LNDFH, Lacto-N-difucosyl-hexaose; LNT, lacto-N-tetraose; pLNH, p-Lacto-N-Hexaose; LNFP, lacto-N-fucopentaose; LNnT, lacto-N-neotetraose; LNT, lactoN-tetraose; LST, sialyl-lacto-N-tetraose; 2′-FL, 2′-fucosyllactose; 3-FL, 3-fucosyllactose; 3′-SL, 3′-sialyllactose; Hex, hexose; HexNac, N-acetylhexosamine; Fuc, fucose and NeuAc, N-acetylneuraminic acid. Fucosylation was further investigated to determine difference in abundance of mono, di, tri, and tetrafucosylation (based on the number of fucose residues).
