# Supplementary figures and images for: Comprehensive Preterm Breast Milk Metabotype Associated with Optimal Infant Early Growth Pattern
Source: Nutrients. 2019 Feb 28;11(3):528. doi: 10.3390/nu11030528 (PMC6470768; doi:10.3390/nu11030528)

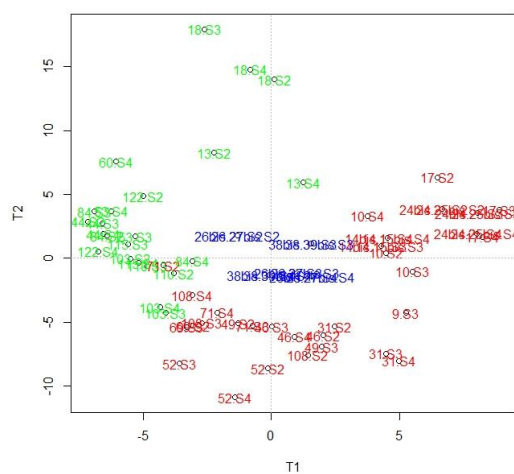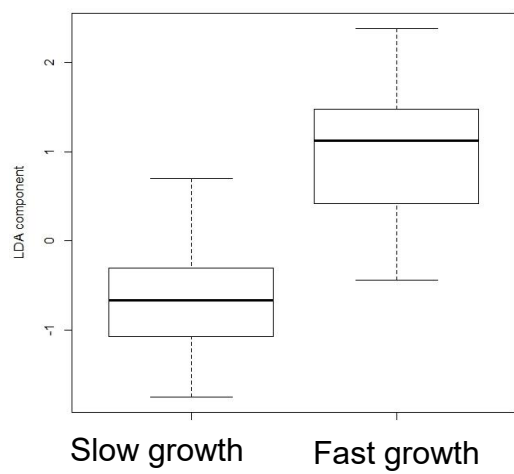

Supplement: Supplementary file 1 [file nutrients-11-00528-s001.pdf]
